# Tuberculous Empyema Necessitatis in a 40-Year-Old Immunocompetent Male

**DOI:** 10.1155/2016/4187108

**Published:** 2016-07-31

**Authors:** Farhang Babamahmoodi, Lotfollah Davoodi, Roya Sheikholeslami, Fatemeh Ahangarkani

**Affiliations:** Antimicrobial Resistance Research Center, Department of Infectious Diseases, Mazandaran University of Medical Sciences, Sari, Iran

## Abstract

Empyema necessitans (EN) is a kind of empyema that diffuses to extrapleural space and can involve chest pain. Tuberculosis (TB) is the most common cause of EN. This disease can be found in both immunocompromised and immunocompetent individuals but is usually seen in the immunocompromised individuals. Because of long duration and ambiguous symptoms of the disease, diagnosis can be hard. The disease can be treated both medically and surgically. Missing the disease can lead to undesirable effects on patient's condition and health care setting. This problem can be seen in endemic area in which controlling of TB is hard. Report of the disease in local health care center for desirable treatment and health maintenance is necessary. We explained a rare case of pulmonary TB in a patient that was healthy in other fields and just showed the minimum systemic symptoms. The patient came with a mass in lower part of back of chest cage, with a mild pain. The imaging survey showed EN. Smear and Ziehl-Neelsen stains from subcutaneous aspiration were positive for TB. This case showed importance of clinical view and awareness of this silent but serious disease in endemic area especially for TB.

## 1. Introduction

Empyema necessitans (EN) is a kind of empyema that diffuses to extrapleural space and can involve chest pain. TB is the most common cause of EN. EN usually presents as a single mass with or without pain on chest wall; diagnosis is based on clinical view and radiologic imaging and confirmation is by smear, culture, and PCR from fluid aspiration. The treatment is combination of drainage and standard anti-TB treatment.

## 2. Case Report

The patient was a 40-year-old male shop keeper with complaint of pain and a mass in right side of chest cage. The symptoms started 20 days ago in right lower part of back of chest which had increase in size gradually, with cough, sputum, fever and chills, pleuritic chest pain, and dyspnea. The patient indicated loss of appetite and loss of weight of approximately 7 kg in recent 20 days.

*Past Medical History*. There was no history of diabetes mellitus, hypertension, surgery, or blood infusion and there was also no history of respiratory problems. 

*Familial History*. The patient was married and his family relations had no recent infectious disease. 

*Social History*. The patient has been smoking 3-4 cigarettes per day for 15 years. He has been addicted to opium for 14 years, both orally and by inhalation, and he had a history of 2 years in jail. 

**Figure 1:**
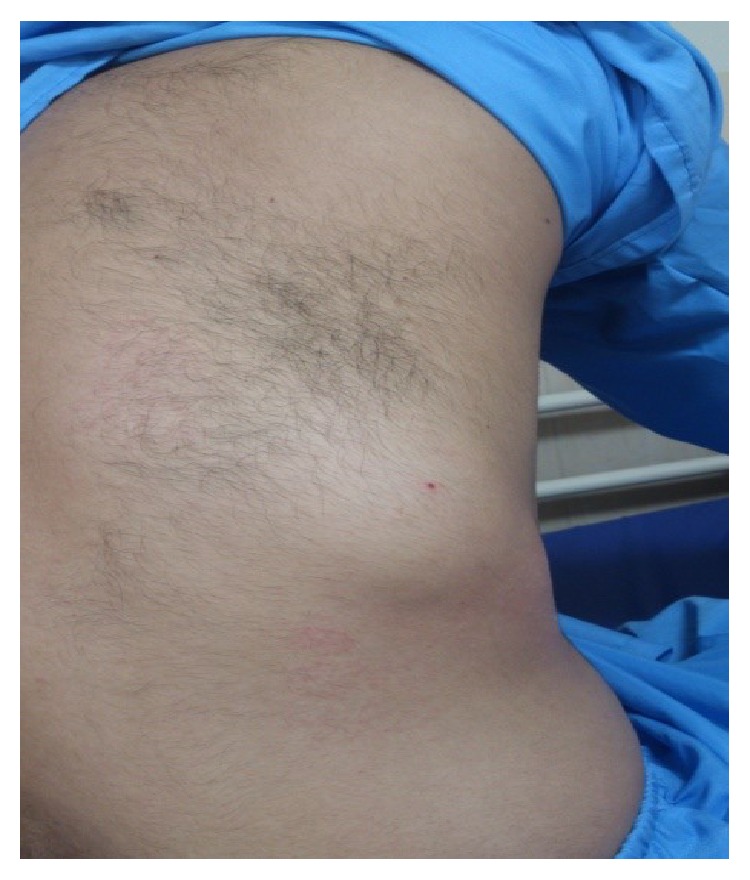
Elliptical bulge of 8*∗*4 cm in lower part of back of right hemithorax.

*Physical Examination*. Vital signs were stable. No specific disorders were found in physical examination of head and neck, ear, nose, and lymphatic system. Heart sounds were normal. There was elliptical bulge of 8*∗*4 cm in lower part of back of right hemithorax (Figure 1). There was no fluctuating in palpation but there was mild tenderness. There was wheezing heard in lower part of right chest cage. But no rale and decrease in sound were found.

**Figure 2:**
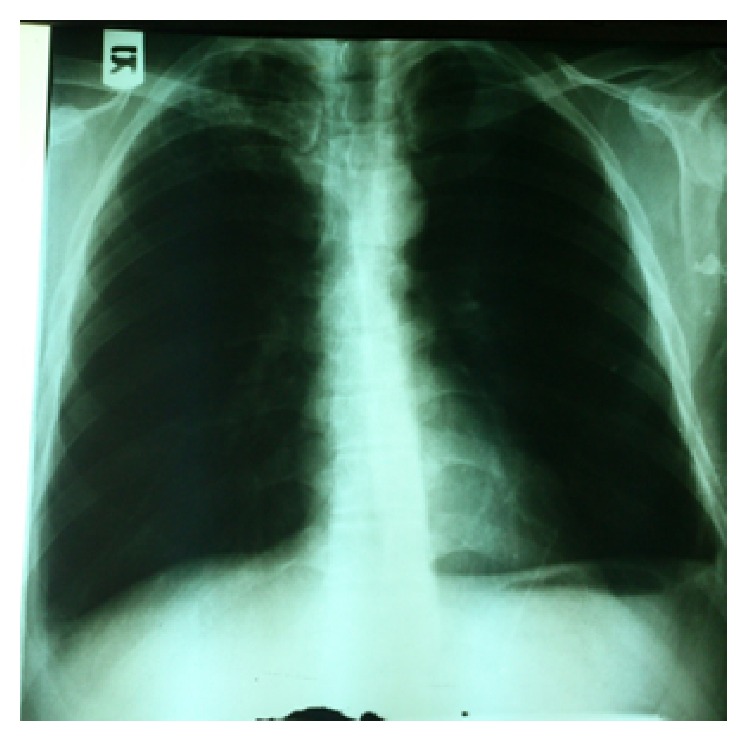
Fibrosis was seen in the apexes especially in the right one.

In chest X-ray, a soft tissue thickness was detected. The right costophrenic angle was blunt but with or without significant fluid. Left diaphragm became flat which could be possibly because of an old problem and there was no subpulmonic effusion. Fibrosis was seen in the apexes especially in the right one (Figure 2).

**Figure 3:**
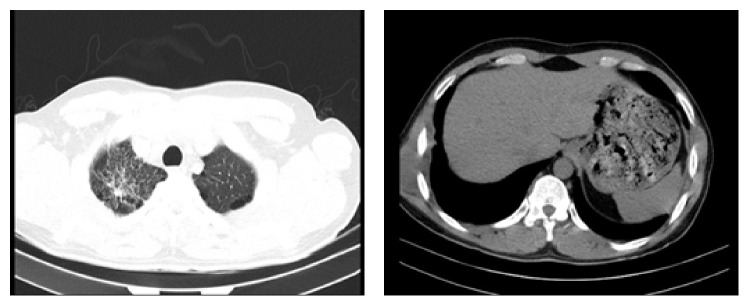
Parenchymal reticular densities were sign of an active process.

In CT scan with and without contrast, fibrosis was seen in apexes especially the right lung which leads to parenchymal deformity and bullous emphysema. Reticular densities were seen in upper part of right lung. Free fluid did not exist. Skin and under skin fact were normal. Ribs and muscles were not involved in the swollen soft tissue area; pleural involvement was obvious. Parenchymal reticular densities were sign of an active process (Figure 3). 

*Laboratory Tests*. CBC, LFT, and electrolytes were normal (PPD: 25 mm, CRP: ++, and ESR: 65 units).

**Figure 4:**
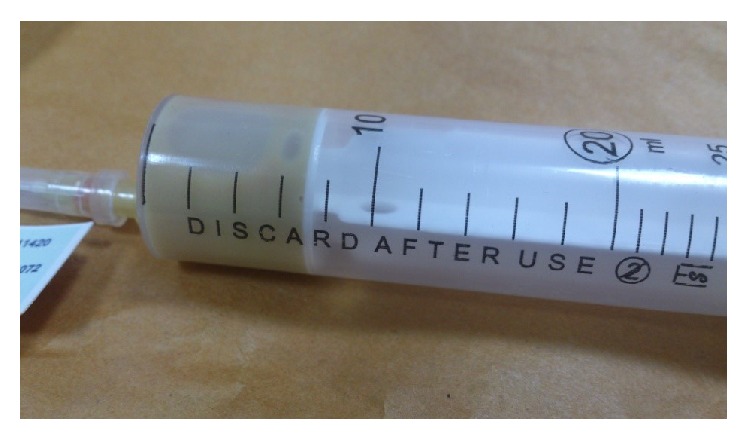
Aspirated fluid.

**Figure 5:**
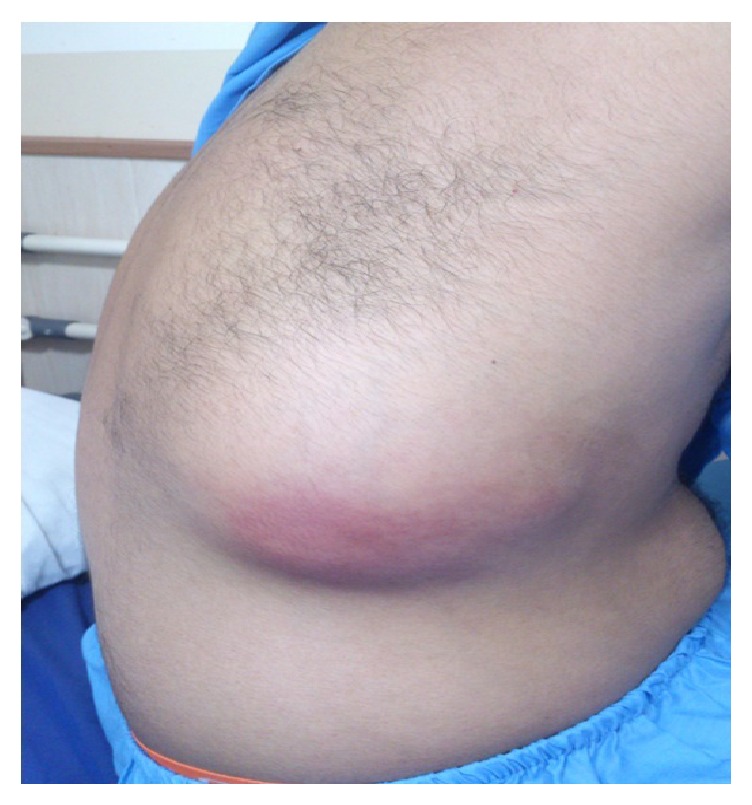
After 40 days of anti-TB treatment.

Subcutaneous fluid tap was done. Aspirated fluid was full of thick PUS, which was 3+ basil acid fast in Ziehl-Neelsen stain (Figure 4). Culture of* Mycobacterium tuberculosis* was positive too. DOTS treatment regime program was activated. After 40 days of anti-TB treatment, the patient came back with worse conditions (Figure 5). So the surgery was performed and then medical treatment was continued. After 6 month of anti-TB treatment, the patient was completely cured and after one year relapse did not occur.

## 3. Discussion

Extrapulmonary TB consists of 15% of total TB. Chest wall abscesses are seen in less than 15% of musculoskeletal TB [1, 2]. Abscesses are due to chronic inflammation of pleural space, which at first start as an empyema and then lead to bronchopleural fistula that causes the leakage of substance to the chest wall. In this condition it is called empyema necessitans, a rare complication in which pus makes its way through soft tissue to the skin [3].

This inflammatory process can remain with unspecified clinical symptom for years, which is seen in both immunocompromised and immunocompetent individuals [4, 5]. These problems are usually described as a single mass without pain in chest wall [6, 7]. Sometimes the patients can have multiple masses that can be painful [4, 8, 9]. EN can distract bones, muscle, soft tissue, and especially the ribs seriously; it is possible to show no symptoms until obvious necrosis occurs [10, 11].

Lung CT scan is pathognomonic in diagnosing EN. The sign is connection of pleural effusion with extrapleural mass of chest wall [12]. CT scan findings are as follows: ribs damage, pleural thickening, and/or its calcification [13]. Rib thickening is seen usually [4]. Sometimes tracheobronchial fistula is seen which can be hardly diagnosed by CT scan [14]. CT scan also can give us some information about function of lung parenchyma. If the typical changes in the CT scan are found, histopathologic analysis would be indicated [15].

Sonography is a cost-benefit way that can show subcostal necrosis and associated soft tissue abscesses [13].

Diagnosis without surgery is usually difficult, because acid-fast bacillus smear and culture, FNA, and PCR have false negative results [16]. PCR is a quick diagnostic way, and we can rely on it for our treatment without culture result. However, this technique is not available everywhere. Surgical histologic samples do have false negative results, and only 20% of cases lead to definite diagnosis [17]. Our patient had positive acid-fast smear.

The most common cause of EN is bacterial TB. Other differential diagnoses are* Staphylococcus aureus*, streptococcal infection, and actinomycosis [18, 19]. EN has to hold in account pulmonary aspergilloses secondary syphilis and typhoid too. Recent travel to endemic area and residency in this part are some important points for medical history. Noninfectious disease such as lymphoma primary lung neoplasm should be considered. Tissue biopsy can rule out these causes even if definite diagnosis of TB is not accessible [20, 21].

Treatment of EN is combination of surgical treatment and medical treatment. Surgery is needed to expand the collapsed lung and to open the intercostal spaces [2]. Many surgeons suggest the aggressive removal of involved tissues [22]. Patient's refusal of complete surgery is effective in possible relapses and is responsible for 2 months of further admission. Recurrent symptoms are maybe because of relapse of inflammatory response due to incomplete drainage of infectious tissue and this problem can be solved by primary aggressive surgery [23].

Chest wall abscesses that involve the ribs need extensive debridement. Removal of all involved tissues such as bones and cartilages is an assured and safe approach but sometimes spreads the infection [1, 24]. Decortication can better the lungs' function significantly [25].

Surgery's mortality rate is about 5%. Relapse of infection is due to incomplete excision of ribs or infected pleura, which can take place 10 years after the surgery. These patients should be under close observation for years [26, 27]. In children, medical treatment without surgery can be a choice [8].

Anti-TB treatment is necessary for prevention of relapses without consideration of surgery usually [28]. Most of the export individuals suggest 6 months to 1 year of treatment with anti-TB agent [1, 27]. If the ribs are infected, removal of the ribs with anti-TB drugs for 1 year is necessary [29, 30].

## 4. Conclusions

EN is an empyema that diffuses to extrapleural spaces and involves chest pain.* Mycobacterium tuberculosis* is the most common cause of EN [31]. This empyema can cause tuberculosis cold abscess of chest wall which is rare but curable. Treatment is surgery and anti-TB chemotherapy. History of this patient shows that TB should be considered as a differential diagnosis for chest wall painless masses, in endemic area, especially even if signs of pulmonary TB or systemic inflammation are absent or minimum. To label this diagnosis we have to have a strong clinical view.
